# Numerical modeling of the distribution of virus carrying saliva droplets during sneeze and cough

**DOI:** 10.1063/5.0018432

**Published:** 2020-08-11

**Authors:** Mohammad-Reza Pendar, José Carlos Páscoa

**Affiliations:** Department of Electromechanical Engineering, C-MAST (Center for Mechanical and Aerospace Sciences and Technologies), University of Beira Interior, Covilhã, Castelo Branco 6201-001, Portugal

## Abstract

Violent respiratory diseases, i.e., coronavirus (COVID-19), spread through saliva in coughs and sneezes or are even exhaled in the form of microbial pathogen micro-droplets. Therefore, in this work, a comprehensive fully coupled Eulerian–Lagrangian method has been applied for infection control, thus leading to a deeper understanding of the saliva-disease-carrier droplet transmission mechanisms and also of their trajectory tracking by using the OpenFOAM package. This model determines the droplet–air interactions, the breakup process, and turbulent dispersion forces on each micro-droplet that is expelled within the respiratory tract in a correct way. By examining a broad range of initial velocities, size distributions, injection angles of saliva micro-droplets, and mouth opening areas, we predict the maximum opening area that can be driven by micro-droplets. One important contribution of this work is to present a correlation for the length and width of the overall direct maximum reach of the micro-droplets, driven by a wide range of mild coughs to intense sneezes. Our results indicate that the movement of the expelled droplets is mainly influenced by their size, angle, velocity, and environmental factors. During a virus crisis, like COVID-19, this paper can be used to determine the “social distance” between individuals to avoid contamination, by inhaling or touching their bodies, due to these saliva-disease-carrier droplets in sneezing, at various social distance positions such as face-to-face, meeting standing, and near equipment. The safe distance must be increased to around 4 m during a sneeze. By wearing a face mask and by bending the head during a sneeze as a protective action, we can reduce the contamination area to one-third and three-quarters, respectively. Furthermore, the dispersion of the film of the expelled saliva micro-droplets and the spatial relationship between the subjects, which affects the airflow inside the room, are also analyzed in detail.

## I. INTRODUCTION

A considerable number of respiratory infectious diseases such as coronavirus (COVID-19), severe acute respiratory syndrome (SARS), Spanish flu (H1N1), and influenza are transmitted among humans through micro-droplets and airborne routes, endangering their lives (WHO, 2002). In 2019–2020, the coronavirus epidemic reached more than 10 × 10^6^ infections and is known as the twenty-first century’s most pandemic disease. In our modern world, respiratory infectious diseases such as COVID-19 would cause many deaths, economic losses, and social disruption (Li *et al.*, 2020). A deeper understanding of the dispersion and transmission mechanisms of saliva-disease-carrier droplets during our respiratory activity such as sneezes and coughs is needed to control these infectious pandemics. Understanding more about aerosolization and virus spreading is an essential issue for carrying out preventive measures such as social distancing, indoor ventilation, and face mask-wearing. Respiratory infectious diseases can be transmitted through direct contact with the expelled micro-droplets (Leder and Newman, 2005) or by indirect connections that occur when the micro-droplets have been deposited on a surface (Chao *et al.*, 2008). Yan *et al.* (2018) concluded that the flu virus can be transmitted through an emanated droplet during talking or even by breathing. While the transmission mechanisms are still under debate, it is commonly accepted that respiratory droplet transmission induces a continued circulation of the influenza virus among individuals (Richard *et al.*, 2020). Beans (2020) confirmed that SARS-CoV-2 can be spread via aerosolized droplets emitted by exhaling patients, although they noted that they have not been able to confirm if the coronavirus found in ambient air is a viable way to infect humans.

Following a brief introduction to the respiratory infectious diseases, we report thematically studies on the size distribution of saliva micro-droplets during different respiratory activities. Then, some relevant research will be explained in detail. Finally, the overall feature of the current study will be presented.

The findings indicate that the deposition and dispersion mechanisms of the expelled micro-droplets by patients are incredibly dependent on the droplet size (Chao *et al.*, 2008; Wan *et al.*, 2007). A substantial part of literature considers the micro-droplet size distribution of expiratory and saliva during sneeze (Buckland and Tyrrell, 1964; Duguid, 1946), cough (Johnson and Morawska, 2009), talking (Xie *et al.*, 2009; Chao *et al.*, 2009), and breathing (Holmgren *et al.*, 2010; Haslbeck *et al.*, 2010; and Almstrand *et al.*, 2010). They confirmed that in various circumstances, the size distribution of micro-droplets is entirely different. Han *et al.* (2013) reported significant inconsistent values for the sneeze micro-droplet size in comparison with other previous studies. As a reason for these differences, in earlier works, they mentioned common problems that occur, such as the effects of evaporation, the impact of the measuring method, and the experimental equipment error.

The turbulent flow mechanisms created during sneezing are considerably different from other respiratory processes and result in a very large size of the micro-droplets (Johnson and Morawska, 2009), i.e., the size of the micro-droplet in a sneeze is around 18 times larger than that in a cough (Gerone *et al.*, 1966). The velocity of the airflow that is exhaled by a sneeze is also higher than in a cough or breath (Gupta *et al.*, 2009; Gao and Niu, 2006). Studies of the sneeze micro-droplet size are still rare up to now. Xie *et al.* (2009) and Dbouk and Drikakis (2020a) discussed the micro-droplet size and mass during a conversation, cough, and sneeze. They corrected the size distribution of micro-droplets in line with the above-mentioned work of Han *et al.* (2013), since they had noted that it was underestimated in previous research work.

COVID-19 infections transfer at a faster rate due to the higher viral load in the respiratory tract of hosts during breathing and social activities (Bai *et al.,* 2020; Li *et al.*, 2020). The lack of knowledge is evident in guidelines about the social distance and face mask-wearing (Elegant, 2020), mostly based on outdated research (Asadi *et al.,* 2020; Bourouiba, 2020).

Dbouk and Drikakis (2020a) analyzed numerically the effect of wind speeds on the social distancing during the human cough. They found that the saliva droplets travel up to 6 m at a wind speed of 15 kmh^−1^ and a social distance of 2 m is not appropriate for outdoor environments. Blocken *et al.* (2020) studied COVID-19 virus spreading patterns in human subjects’ movement, such as running and cycling, under external wind effects. They proposed that larger social distances (*d* ≥ 1.5 m) must be preserved throughout the external activities. Bourouiba *et al.* (2014) described violent expiratory fluid dynamics of sneeze and cough. They predicted the pathogen range and introduced the fall out model. Zhao *et al.* (2005) reported a numerical analysis for the droplet distribution during the sneezing process. They confirmed that droplets traveled longer distances during sneezing than breathing, and this poses an increased risk of human body SARS infection. Bhardwaj and Agrawal (2020) investigated the drying time of respiratory droplets expelled from an infected COVID-19 person by evaluating the droplet contact angle, volume, temperature, and environmental humidity. They predicted that drying time is a crucial factor when infecting another subject, and a diffusion limited evaporation approach for a sessile droplet on a partially wetted surface was implemented. Zhu *et al.* (2006) demonstrated that the transport characteristics of the saliva droplets due to coughing in a calm indoor atmosphere are able to change with their size and can travel further than 2 m. They further observed that the inertia and gravity of droplets with *d*_*p*_ ≤ 30 *µ*m in diameter are negligible. They found that droplets with size between *d*_*p*_ = 50 *µ*m−200 *µ*m and *d*_*p*_ ≥ 300 *µ*m are significantly affected by gravity and inertia. Wan and Chao (2007) investigated the importance of the ventilation system on the infection risks by expiratory droplets. They revealed that unidirectional-upward and single-side-floor ventilation systems are more useful to mitigate the effect of small and large droplet distributions, respectively. Dbouk and Drikakis (2020b) studied the fluid dynamics of respiratory droplets induced by a mild incident of coughing around a face mask filter. They investigated the interaction modes, i.e., rebound, stick, and penetration of saliva droplets onto the fibrous porous surface of the mask. They found that the travel distance of droplets by wearing a mask is about half of the distance when on the naked face, and this distance becomes larger during incremental cough cycles. To have an accurate simulation, selecting an appropriate turbulence model is a vital question (Pendar and Pascoa, 2019b). The large eddy simulation (LES) approach is suitable for capturing flow features and for analyzing the internal flow principle, which is used in the current work (Roohi *et al.*, 2016; Kolahan *et al.*, 2019; and Pendar and Roohi, 2015).

In our study and by using the OpenFOAM package, we modeled the size, velocity, and spatial distribution of the expelled micro-droplet through sneezing and coughing. These findings are useful for effective prevention of infectious droplet-borne and airborne diseases, in particular, coronavirus (COVID-19), by identifying the transmission processes in different places such as hospitals.

## II. GOVERNING EQUATIONS

### A. Continuous phase

For the carrier bulk multiphase flow, the mathematical formulations include a continuous and discrete phase. The compressible Navier–Stokes equations, in conjunction with a large eddy simulation (LES) turbulence model, are applied for modeling the flowfield. The continuity and momentum equations used in the LES model with Favre-averaging operation are defined as follows:

$$\frac{\partial \rho }{\partial t}+\frac{\partial \left(\rho \bar{u_{j}}\right)}{\partial x_{j}}=0,$$ (1)

$$\frac{\partial \left(\bar{\rho }{\bar{\text{u}}}_{j}\right)}{\partial t}+\frac{\partial \left(\bar{\rho }{\bar{\text{u}}}_{i}{\bar{\text{u}}}_{j}\right)}{\partial x_{j}}=-\frac{\partial \bar{p}}{\partial x_{i}}+\frac{\partial {\bar{\sigma }}_{ij}}{\partial x_{j}}-\frac{\partial \tau_{ij}}{\partial x_{j}}+S.$$ (2)

*S* is used to denote other forces, such as surface tension and gravity, which are acting on the fluid. The subgrid-scale (SGS) stress tensor (*τ*_*ij*_) is modeled by employing an eddy-viscosity approach,

$$\tau_{ij}\approx \rho \left(\bar{u_{i}u_{j}}-{ū}_{i}{ū}_{j}\right),\;\tau_{ij}=\frac{2}{3}\bar{\rho }kI-2\mu_{k}\bar{S_{ij}},$$ (3)

$$\bar{S_{ij}}=\frac{1}{2}\left(\frac{\partial \bar{u_{i}}}{\partial x_{j}}+\frac{\partial \bar{u_{j}}}{\partial x_{i}}\right).$$ (4)

Here, *S*_*ij*_ is defined as a rate-of-strain tensor for the resolved scale. We employed the “one equation eddy-viscosity model” (OEEVM) subgrid-scale (SGS) in the current work. To obtain the turbulent kinetic energy (*K*), the OEEVM is solved as follows:

$$\partial \left(\bar{\rho }k\right)+\nabla \cdot \left(\bar{\rho }ku\right)=-\tau_{ij}\cdot \bar{S_{ij}}+\nabla \cdot \left(\mu_{k}\nabla k\right)+\bar{\rho }\varepsilon ,$$ (5)

$$\varepsilon =c_{\varepsilon }k^{3/2}/\Delta .$$ (6)

The SGS turbulent viscosity, *μ*_*k*_, is calculated from

$$\mu_{k}=c_{k}\bar{\rho }\Delta \sqrt{k}.$$ (7)

Here, *C*_*ε*_ and *C*_*k*_ are set as 1.048 and 0.094, respectively, in the present implementation. The governing equations that are used in the current study are mentioned in detail in Pendar and Páscoa (2019a). The saliva micro-droplets interact with the injected respiratory airflow, from the mouth, and with the ambient airflow.

### B. Discrete phase

The discrete phase that refers to the processes of dispersion of saliva droplets throughout the computational domain is solved as a series of differential equations using a Lagrangian approach. Through these differential equations, we compute the velocity, mass, and position of each individual droplet in each time step. The following equation for the saliva micro-droplet trajectory considers the effect of gravity, Stokes drag, added-mass force, and pressure variation:

$$\begin{array}{ccc} m_{p}\frac{\partial {\vec{u}}_{p}}{\partial t} & = & {\vec{F}}_{G}+{\vec{F}}_{D}+{\vec{F}}_{M}+{\vec{F}}_{P}\\ & = & \left(\rho_{P}-\rho_{f}\right)V_{P}\vec{g}+\frac{3}{4}C_{d}\frac{\rho_{f}}{\rho_{P}}\frac{m_{P}}{2R_{P}}\left|\left({\vec{u}}_{f}-{\vec{u}}_{P}\right)\right|\left({\vec{u}}_{f}-{\vec{u}}_{P}\right)\\ & & +\;\frac{\rho_{f}V_{P}}{2}\frac{\partial \left({\vec{u}}_{f}-{\vec{u}}_{p}\right)}{\partial t}+V_{P}\nabla P. \end{array}$$ (8)

Here, ${\vec{u}}_{p}$ and ${\vec{u}}_{f}$ are the saliva particle and fluid velocity vector, respectively. In addition, *ρ*_*f*_ and *ρ*_*p*_ are the air and particles’ density, and *V*_*p*_, *R*_*P*_, and *m*_*p*_ are the volume, the radius, and the mass of the saliva particles, respectively. The values of the drag coefficient *C*_*D*_, which depend on the droplet’s Reynolds number, are given by

CD=24/RePif ReP<1(24/ReP)(1+0.5ReP0.687)if 1≤ReP≤10000.44if ReP>1000, (9)

where

$$\text{R}{\text{e}}_{\text{p}}=\frac{2{\text{R}}_{\text{P}}\left|{\vec{\text{u}}}_{\text{f}}-{\vec{\text{u}}}_{\text{p}}\right|\rho_{f}}{\mu_{f}}.$$ (10)

Here, ${\vec{\mu }}_{f}$ is the fluid viscosity. The final expression of Eq. (8) is written as follows:

$$\begin{array}{ccc} \frac{\partial {\vec{\text{u}}}_{\text{p}}}{\partial \text{t}}\left(1+\frac{\rho_{f}}{2\rho_{p}}\right) & = & \frac{3}{4}\frac{C_{d}}{2{\text{R}}_{\text{P}}}\frac{\rho_{f}}{\rho_{p}}\left|{\vec{u}}_{f}-{\vec{u}}_{p}\right|\left({\vec{u}}_{f}-{\vec{u}}_{p}\right)+\left(1-\frac{\rho_{f}}{\rho_{p}}\right)\vec{g}\\ & & +\;\frac{\rho_{f}}{2\rho_{p}}\frac{\partial {\vec{u}}_{f}}{\partial t}+\frac{\nabla P}{\rho_{p}}. \end{array}$$ (11)

Disease transmission by larger saliva-disease-carrier droplets is certainly more probable, as mentioned in Wells (1934). The droplet size distribution of droplets is an important factor because of its strong effect on the travel distances; subsequently, it influences the infection risk (Xie *et al.*, 2009). The breakup approach is one of the most important sub-models to be considered during the coughing simulation. The primary breakup process in the current work is implemented through the Rosin–Rammler approach. This procedure in a person’s mouth is modeled by seeding different ranges of droplet radii by invoking a presumed probability density function (PDF). In OpenFOAM, the Rosin–Rammler PDF is expressed as

$$f\left(r\right)=\frac{qr^{q-1}}{{\bar{r}}^{q}}\exp\left[-{\left(\frac{r}{\bar{r}}\right)}^{q}\right],$$ (12)

where *q* and $\bar{r}$ are the exponential factors and average radius, respectively, which are based on the saliva injection flow rate as an input parameter for the considered seeding droplet *N*_*droplet*_. An exponential factor is set as *q* = 2.56 in this work. The average radius ($\bar{r}$) of the droplet is reported in Table II(a). The modified TAB breakup model (Tanner, 1997), which is a developed version of the original TAB model, is applied as a secondary breakup model. In this model, there is a proportional relation between the droplet number and its rate of production. In addition, the balance of energy among the parent and produced droplets results in an expression for the velocity component of the newly produced droplet. Here, the Ranz and Marshall (1952) heat transfer model is used to evaluate the reduction in saliva droplet mass due to evaporation. Then, the temperature of the droplets is computed by solving energy and enthalpy equations, which are presented in detail in Dbouk and Drikakis (2020a). Here, the developed trajectory collision model (Nordin, 2001), which is available in the OpenFOAM code, is implemented. The model considers the droplet local interaction procedure with the mask, which is used here, as described in Dbouk and Drikakis (2020b). Penetration, rebound, and stick interaction modes are a function of the maximum droplet diameter, Weber number, and Laplace number, respectively; all of them happen during this phenomenon. We will explain in detail the above behavior in Sec. IV for our specific case.

## III. NUMERICAL SETUP

**FIG. 1.**
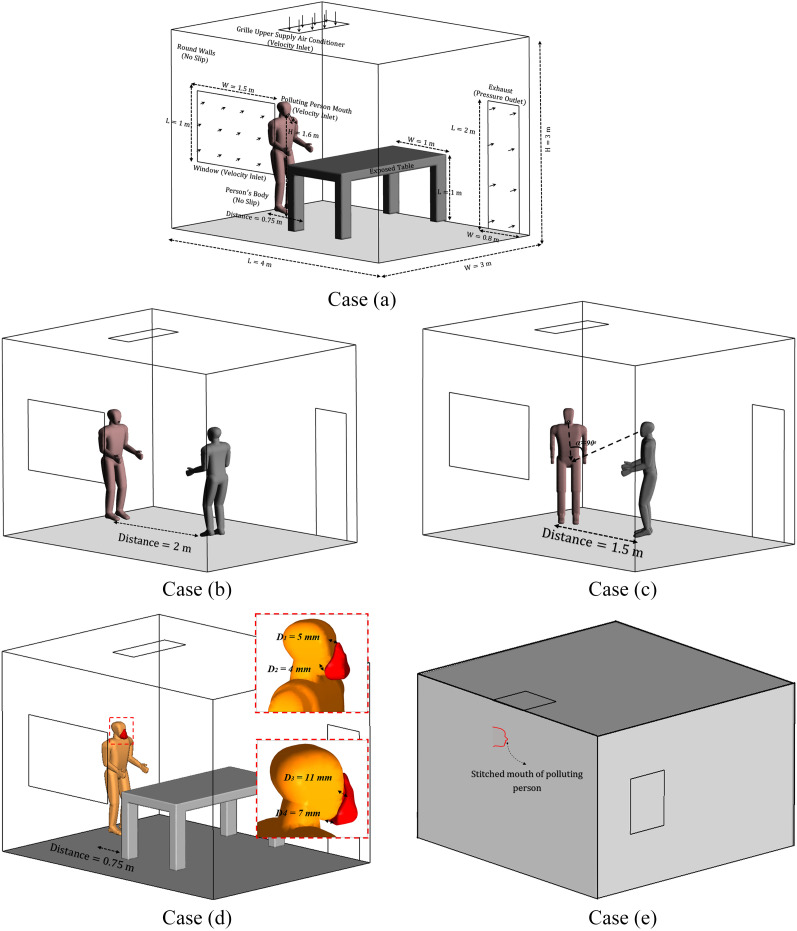
The computational domain dimensions and boundary conditions. Case (a): subject facing a table at 0.75 m distance. Case (b): face-to face position at 2 m distance. Case (c): meeting position at 1.5 m distance. Case (d): subject facing a table while wearing a face mask. Case (e): subject standing close to the side wall.

In the current study, we examined various configurations of full-scale polluted persons and exposed individuals, or equipment, at different lateral distances. The distances for the following cases were selected to assess the suggested social distance by the Centers for Disease Control (CDC) and World Health Organization (WHO) during normal activities of the subjects [CDC, 2020; WHO, 2020]. Figure 1 displays the computed computational domain in combination with all dimensions and boundary conditions. A full-scale room with the dimensions of X × Y × Z = 4 × 3 × 3 m^3^ is used as an indoor environment for the current simulation. This room is ventilated via an air conditioner on the ceiling and a window and door on the side wall. A polluted person is shown in a fixed position compared to another person or equipment in other locations:

case (a): the position near the table (L = 0.75 m);
case (b): face-to-face position (L = 2 m);
case (c): meeting standing position (*α*° = 45 and L = 1.5 m);
case (d): face mask-wearing by the polluting person at case (a) (L = 0.75 m); and
case (e): a polluting person standing outside the room and just the mouth is stitched onto the side wall and sneezing inside the room.

Face mask tight-fitting is a critical factor that affects the mask performance. As indicated in Fig. 1(d), the minimum and maximum distance between the mask and face are here considered 4 mm and 11 mm, respectively. We set the temperature of the human’s mouth and environment at 34 °C and 20 °C, respectively. The polluting person’s mouth altitude from the floor is considered to be about 1.6 m. The expelled saliva’s initial total mass is estimated to be 15 mg. Here, the saliva is considered as a Newtonian fluid with a density of 998 kgm^−3^; however, it is certainly a more complex fluid. We applied a constant inlet velocity boundary condition for the polluting person’s mouth, window, and air conditioner with a turbulence intensity of 20% in the specified direction. The velocity of the respiratory airflow and saliva micro-droplets that are injected from the mouth is varied between 6.3 ms^−1^ and 22.3 ms^−1^, typical of various statistically representative cough and sneeze, as reported in the experimental data of Chao *et al.* (2009). The air conditioner and window induce an airflow with velocities set at 0.6 ms^−1^ and 0.2 ms^−1^, respectively. At the exhaust door, an outlet pressure boundary condition is used. We defined all remaining boundaries (human bodies, other walls, and floor) as a no-slip wall boundary condition, applying wall functions to implement the turbulent boundary layer properly.

**FIG. 2.**
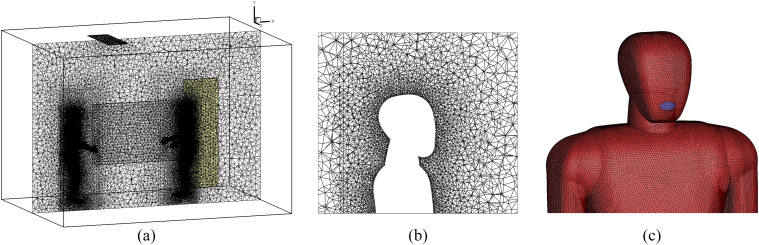
Computational grid with specific refinement near the mouth. The total cell count is about 5.1 × 10^6^ cells. (a) Z-axis, (b) close-up view near the head, and (c) body surface.

**FIG. 3.**
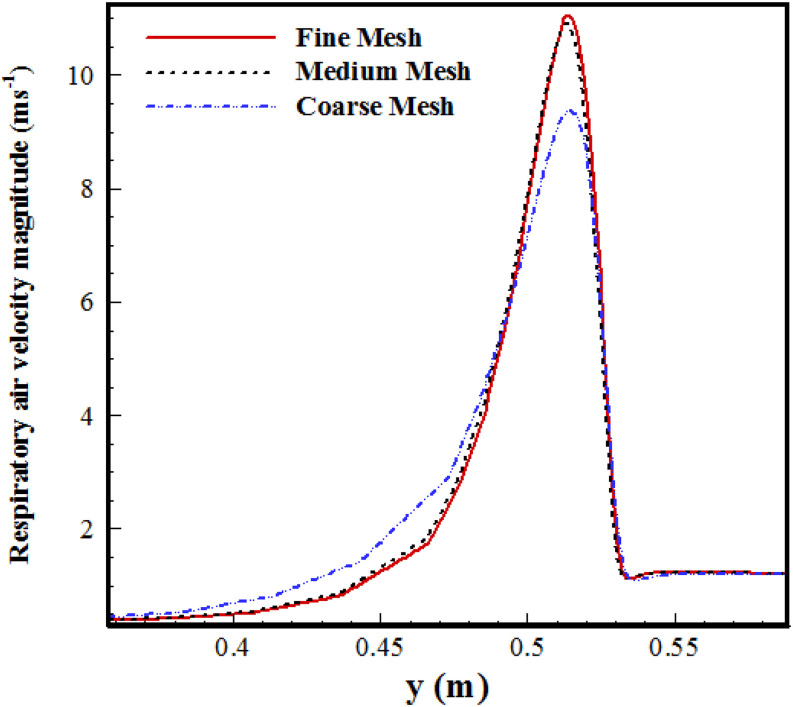
Velocity magnitude compared between coarse, medium, and fine grids near the mouth (at a distance of 0.03 m near the mouth).

Figure 2 shows the three-dimensional (3D) computational grid that we used in our simulation with a total cell number of 5.1 × 10^6^ (medium grid). We generated a mesh comprising tetrahedral, non-uniform, and unstructured cells because of the complex geometry of the human body. Table I presents the quality of three different grids, e.g., maximum skewness, aspect ratio, and boundary layer size. The grid size is progressively increased outward of the body surface, where the variations in flow are proportionally small. Since the accuracy of results strongly depends on the mesh size, a dense mesh near the head, especially around the mask region, is used. This method helps to save the computational cost by decreasing the total cell numbers for the present complex geometry. The applied computational grid shows a high resolution, in particular, close to the body surface and on mouth-print, around 1.9 mm, which is coarsened in the outward direction with a ratio of 1.15. The selection of this grid size has been conducted based on analyzing the fluid velocity (U_f_). Figure 3 compares the velocity magnitude profile sampled at a vertical line close to the mouth with the coarse, medium, and fine grid.

**TABLE I.** Element description of the coarse, medium, and fine grid for the “face-to-face” case.

	Fine	Medium	Coarse
Description	grid	grid	grid
Total element number	6 842 897	5 123 271	2 234 562
Boundary layer element size (mm)	1.5	1.9	3.8
Volume element size (mm)	1.2–15	1.4–19	3.2–38
Maximum aspect ratio	23.3	26.6	37.2
Maximum cell skewness	0.972	0.938	0.915

Table II presents the distribution of saliva micro-droplets produced using the Rosin–Rammler method with a minimum, average, and maximum diameter, in accordance with the approximately equivalent range of values used in Blocken *et al.* (2020), Han *et al.* (2013), and Dbouk and Drikakis (2020a). Based on the experimental results (Han *et al.,* 2013), one single sneeze can produce more than O (10^4^) saliva droplets, and we considered the same range in our numerical simulation. Han *et al.* (2013) considered the forward velocity for a sneeze and cough as 20 ms^−1^ and 10 ms^−1^, respectively. Wells (1934) estimated that about 100 *µ*m is the critical size, which can be recognized as a boundary between large and small droplets. Various cases are also considered with a broad range of coughing/sneezing horizontal angles and mouth-printed areas in the present simulations [Table II(b)]. Our simulation is implemented under the framework of the open-source Computational Fluid Dynamics (CFD) code “OpenFOAM” (Jasak, 2009). Second-order schemes are used in discretizing the momentum and continuity equations. For pressure–velocity coupling, the PIMPLE algorithm is used (Pendar and Roohi, 2018).

**TABLE II.** The various situations considered: (a) data for the distribution of micro-droplet diameters and sneezing initial velocity comparisons (*θ*_*inj*_ = 33° and *mouth area* = 314 mm^2^) and (b) comparison of injection angles and mouth areas for different cases.

Bin	*d*_*average*_ (*μ*m)	*d*_min_ (*μ*m)	*d*_max_ (*μ*m)	Δ*V*_*Initial*_(ms^−1^) = 1 ms^−1^		
(a)						
Cases 1–8	90	40	980	6.3–14.3		
Cases 9–16	140	40	980	6.3–14.3		
Cases 17–24	190	40	980	6.3–14.3		
Cases 26–32	240	40	980	6.3–14.3		
Cases 33–40	290	40	980	6.3–14.3		
Cases 41–48	340	40	980	6.3–14.3		
Cases 49–56	390	40	980	6.3–14.3		
Cases 57–64	440	40	980	6.3–14.3		
Cases 65–72	490	40	980	6.3–14.3		
Cases 73–80	540	40	980	6.3–14.3		
		*Moutd area*	*d*_*average*_	*d*_min_	*d*_max_	*V*_*Initial*_
Bin	*θ*_*inj*_	(mm^2^)	(*μ*m)	(*μ*m)	(*μ*m)	(ms^−1^)
(b)						
Cases 1–4	3°−43°(Δ*θ*_*inj*_ = 10°)	314	290	40	980	8.3
Cases 5–8	33°	170, 314, 490, 700	90	40	980	14.3

## IV. RESULTS AND DISCUSSION

Our numerical study revealed how saliva micro-droplets, during coughs and sneezes, can be dispersed in turbulent clouds, precisely the same as in the real condition. We simulate dynamic mechanisms of saliva micro-droplets using the initial parameters, i.e., initial velocity (*V*_*Initial*_), initial size distribution (*D*_*p*_), horizontal injection angle (*θ*_*Inj*_) of the micro-droplets, mouth area, and cloud opening angle (*θ*_*out*_), and this is performed according to that reported in the experimental literature. In addition, the transport of these micro-droplets over a farther distance from the mouth depends on the conditions of the indoor environment, such as complex recirculatory flows of ventilation systems.

Coronavirus transmission occurs in three ways: (a) direct transfer of large droplets expelled at high momentum to the receiver’s conjunctiva, mouth, or nose; (b) physical contact with droplets deposited on the surface and subsequent absorption to the nasal mucosa of the receiver; and (c) inhalation by the recipient of expiratory ejected aerosolized droplet nuclei (Mittal *et al.,* 2020). The suggested social distancing by the Centers for Disease Control (CDC) and World Health Organization (WHO) is around 3–6 feet (0.9 m–1.8 m) [CDC, 2020; WHO, 2020]. The turbulent jet generated with the violent sneeze is spread at a Reynolds number of O (10^4^) (Bourouiba *et al.,* 2014). Competition between gravity, inertia, drag, and environmental forces determines the fate of saliva droplets.

**FIG. 4.**
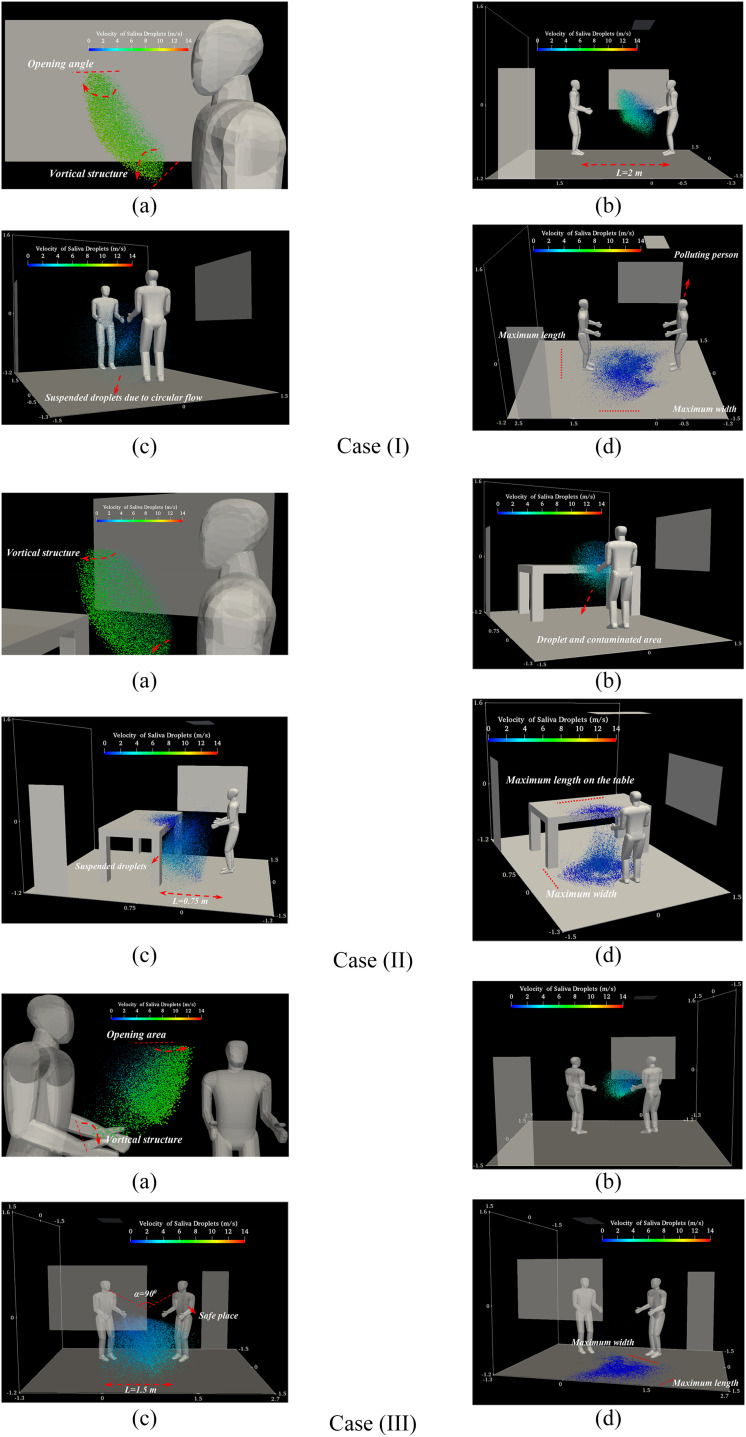
A human sneeze: kinematic visualization of the expelled saliva-disease-carrier droplets for cases (I) face-to-face, (II) meeting standing, and (III) near the table: (a) t = =0.06 s, (b) t = =0.16 s, (c) t = =0.3 s, and (d) t = =1 s. (The initial velocity, total mass, diameter, scale factor, and the number of saliva droplets are *V*_*Initial*_ = 14.3 ms^−1^, *m*_*Saliva*_ = 15 mg, *D*_*average*_ = 360 *μ*m, *D*_min_ = 10 *μ*m, *D*_max_ = 980 *μ*m, *SF*_*Droplet*_ = 15, and *N*_*Saliva*_ = 95 000, respectively.)

Figure 4 shows the saliva micro-droplets kinematics for a complete sneezing cycle, from the mouth to floor, for various social positions. This evolution involves the dispersion, breakup, and deposition process of the saliva droplets during sneezing. First, the droplets expelled from the mouth are mostly affected by the inertia force and are moving along the direction of the initial velocity [frame (a)]. These droplets, first, travel a long distance (0 ms–16 ms). Following the development of a conical jet near the mouth, the droplets entertained a vortical flow [frame (b)]. The droplets’ velocity, with a maximum value of 14 ms^−1^, decreases gradually when they depart from the mouth. During this time, the inertia force gradually decreases and the gravity force controls the larger droplets, while drag and Brownian forces control smaller droplets [frame (c)]. The velocity of the droplets decreases to 2 ms^−1^ after about 16 ms. Therefore, larger droplets immediately fell on the floor and the smaller ones continue to fluctuate and take a lot of time to reach the ground (16 ms to 1 s). Frame (d) shows the virus-laden droplet deposition of the host’s respiratory mucosa in the final stage of transmission. As any virus is able to survive on a surface for hours (Van Doremalen *et al.,* 2020), these droplets are important for the analysis of contaminated surfaces. Larger droplets reach a larger distance and may influence the infection’s intensity and progression. Shorter people are at higher risk since their faces are located on the falling region of the micro-droplet trajectory. The inlet airflow of the air conditioner and window changes the normal path of the low-velocity droplets having a smaller diameter. It increases the flow vortices and intensifies the Brownian movement [frame (c)].

In case (I) (*L* = 2 m), “face-to-face,” the droplets are deposited at a horizontal distance of more than ≈2.8 m away from the mouth. These droplets passed through the opposite person in the area below the chest area. They cannot reach the face of the opposite individual and just can be deposited on the clothes or shoes. In case (II) (*L* = 0.75 m), “person near the table,” the droplets pass entirely over the table, and it is shown that the considered distance is not safe at all. A larger portion of the table surface is polluted due to hitting by the droplets at a high speed of 4 ms^−1^. Finally, in case (III) (*L* = 1.5 m), “meeting position,” the deposition pattern revealed that the preferred length could be considered as a safe distance. For proper visualization of the spatial distribution and deposition of the saliva droplets, their size is magnified by the scale factor of *SF*_*Droplet*_ = 15.

**FIG. 5.**
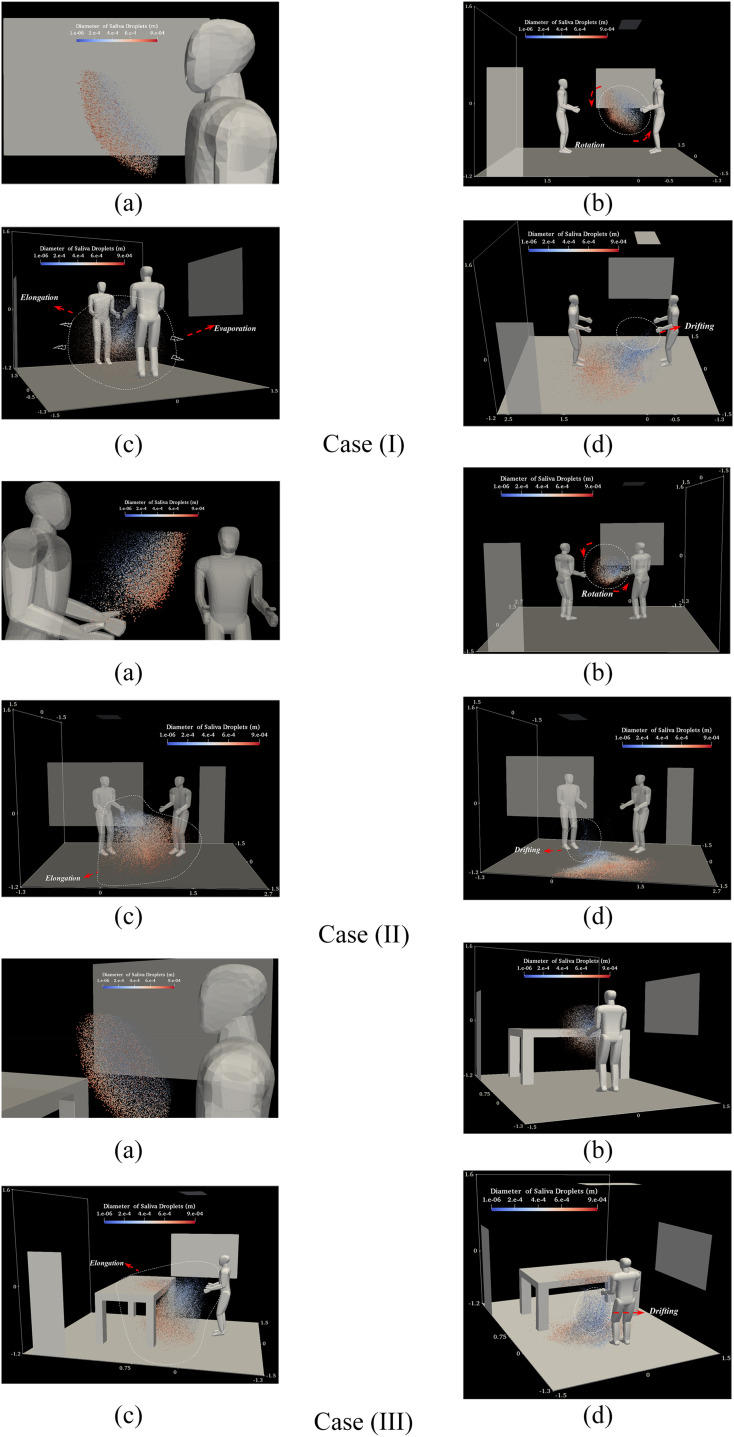
A human sneeze: saliva-disease-carrier droplets’ diameter distribution for diverse cases (I) face-to-face, (II) meeting standing, and (III) near the table: (a) t = 0.06 s, (b) t = 0.16 s, (c) t = 0.3 s, and (d) t = 1 s (the same operational conditions as in Fig. 4).

Figure 5 indicates the distribution of the saliva droplets, which are colored according to their size in the same condition as in Fig. 4. Due to the breakup process, disintegration, and evaporation phenomenon, the values of the Sauter Mean Diameter (SMD) decrease from *D*_32_ = 307 *μ*m to *D*_32_ = 205 *μ*m. Larger droplets, with a diameter larger than the critical size (*D*_*droplet*_ ≈ ≥100 *μ*m), are deposited on the floor or equipment farther and faster by overcoming the inertia and gravity forces on the aerodynamic drag force, before evaporation, but the smaller ones remain airborne and may be transported through the airflow [frames (c) and (d)]. The vortical dynamic cloud has a minor effect on the larger droplet size, but circular flow suspends the droplets with medium and small size. Various saliva droplet kinematic phenomena such as elongation, rotation, and drift are shown in Fig. 5. The saliva droplets are advected in the airflow direction with a slight rotation in the clockwise direction. A few number of droplets disintegrate into a very small size due to the breakup process, and these droplets quickly evaporate. Small droplets, drifting due to their lower effective momentum, became sustainable [frame (c)]. This research shows that the recommended 6 feet (2 m) safe distance is not reliable. For all cases, droplets take about 0.3 s to fall and cross under the human waist.

**FIG. 6.**
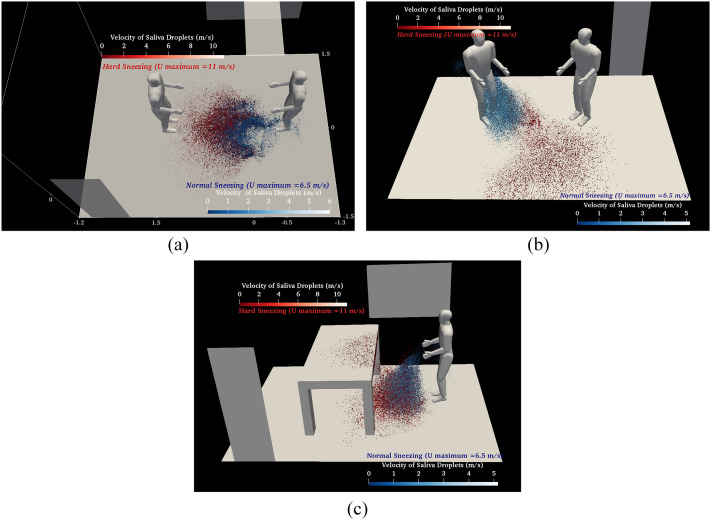
Comparison of the velocity distribution of saliva droplets for two different sneezes—normal (*V*_*Initial*_ = 6.5 ms^−1^) (blue droplets) and hard (*V*_*Initial*_ = 14.3 ms^−1^) (red droplets): (a) face-to-face position, (b) meeting standing position, and (c) near the table position.

Figure 6 compares the flowfield of saliva droplets for two cases with different sneezing ranges and for a different social position: hard sneeze (*V*_*Initial*_ = 14.3 ms^−1^) (red) and normal sneeze (*V*_*Initial*_ = 6.5 ms^−1^) (blue) at *t* = 1 s. It is obvious that the contaminated area, especially the maximum polluted length, due to differences in inertial force is completely different. As shown, the number of drifted and suspended saliva droplets at about 0.6 m above the floor during a normal sneeze is higher than for a hard sneeze since the aerodynamic drag force can easily overcome the inertia and gravity forces. Droplets did not exceed 1 m away from the mouth during a normal sneeze.

**FIG. 7.**
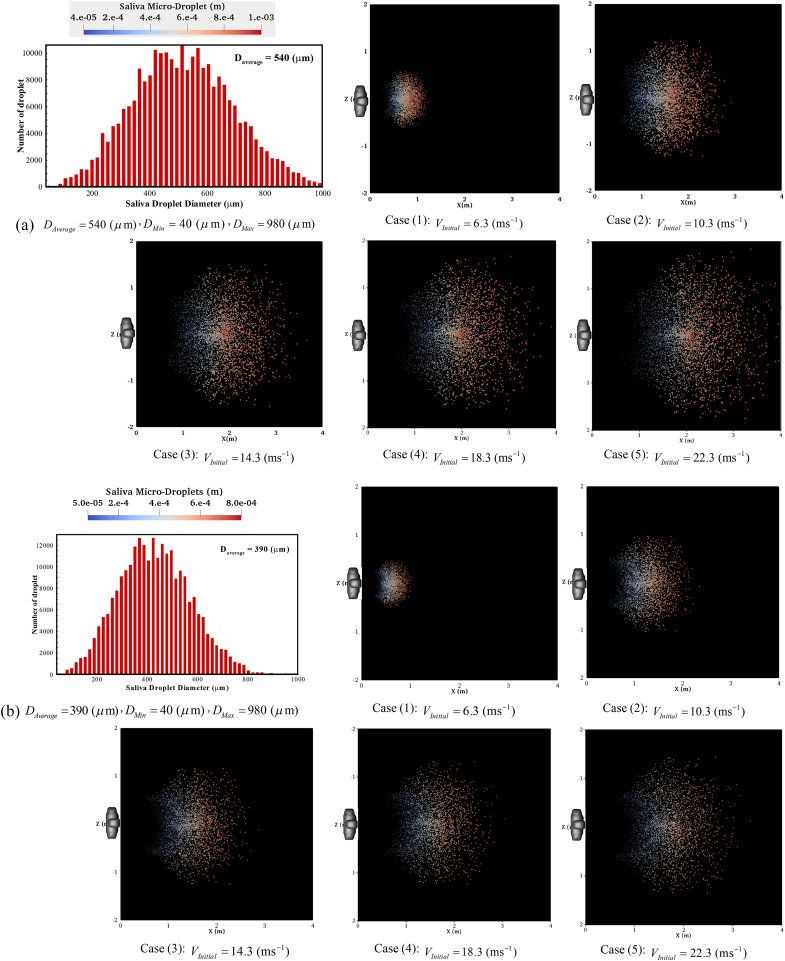

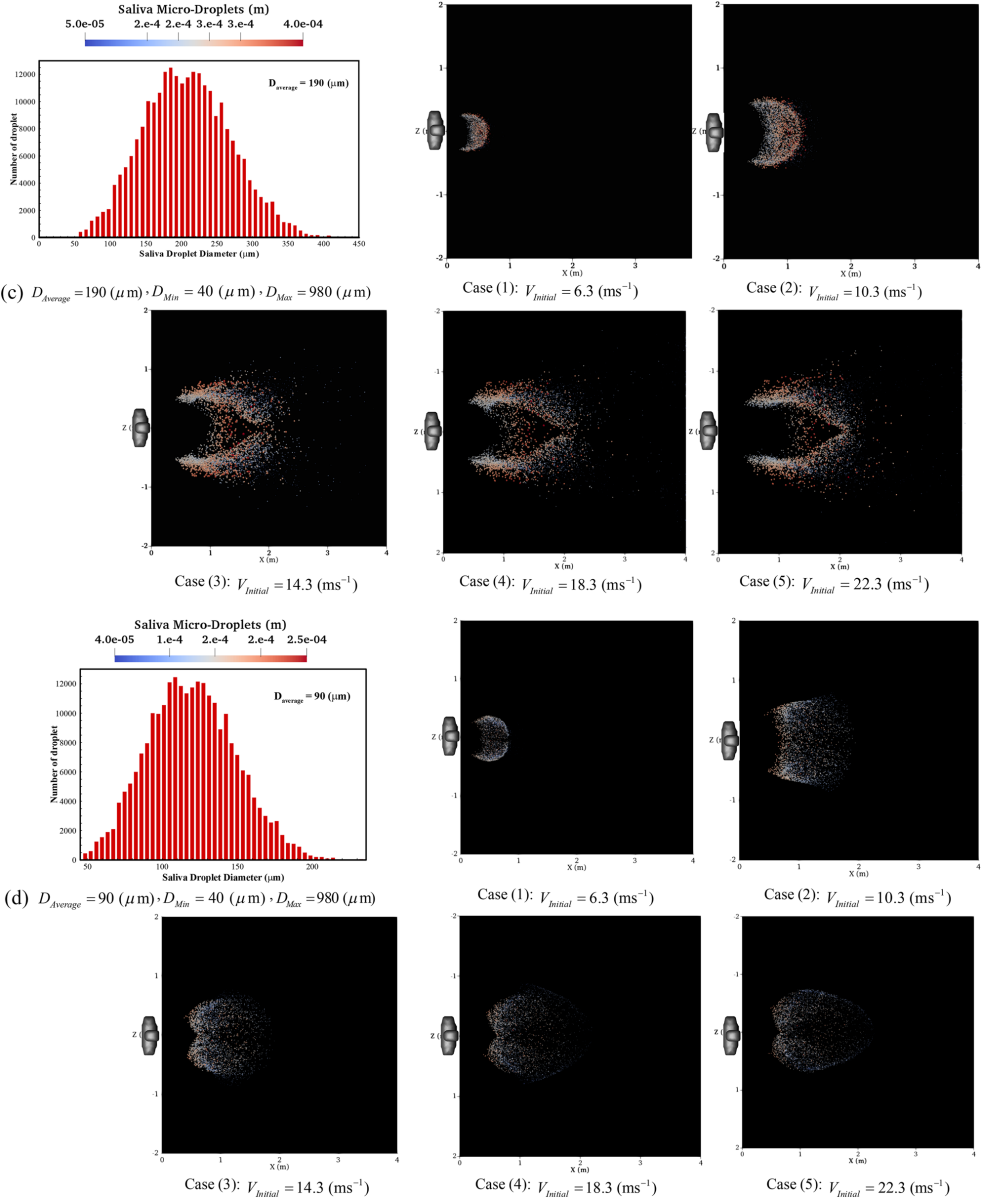
The deposition pattern of micro-droplets of saliva under various operating conditions: (a) *D*_*Average*_ = 540 (*μ*m), (b) *D*_*Average*_ = 390 (*μ*m), (c) *D*_*Average*_ = 190 (*μ*m), and (d) *D*_*Average*_ = 90 (*μ*m).

The maximum horizontal traveled distance of the droplet transmission route is an important factor. Figure 7 shows the pattern of micro-droplets’ deposition for a wide range of initial size distributions and velocities, which is scaled to be visible with naked eyes. In this figure, the maximum deposition area has been assessed precisely. The first frames of each part show a size distribution of droplets that is estimated according to the Rosin–Rammler approach, resulting in various sizes *D*_*Average*_ = 90–540 (*μ*m). The pattern of deposition has clearly changed for various ranges of droplet sizes. For *D*_*Average*_ ≥ 390 *μ*m, they have almost elliptical forms albeit with different sizes. The deposition patterns in these size-ranges clearly demonstrate that the effects of gravity and inertia forces are dominant and the influence of the flowfield is largely diminished. By reducing the size distribution, for example, *D*_*Average*_ ≤ 190 *μ*m, this pattern becomes chaotic, especially for higher initial velocity values that have a disordered pattern with a more elongated shape. The micro-droplets, due to an environmental situation, such as a window or air conditioner inlet flow, have changes in their normal direction before reaching the fall out length. It confirms that through the decrease in the size distribution of droplets, the role of the inertia and gravity forces in determining their trajectory is declining, and the aerodynamic drag force and environmental conditions have a stronger influence. The droplet settling at a farther distance, on the floor, for larger size and long-lived micro-droplets, is settling at a very low speed of around 0.06 ms^−1^.

**FIG. 8.**
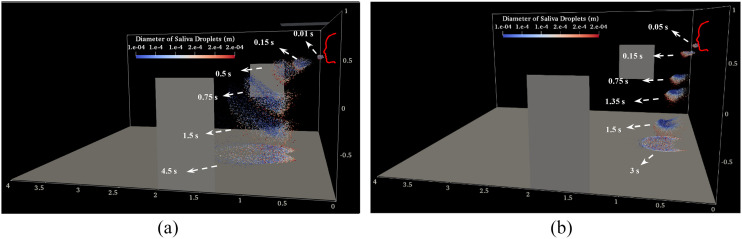
Time evolution of the transport processes of saliva droplets as a function of their diameter during a human sneeze (*D*_*Average*_ = 90 *μ*m): (a) *V*_*Initial*_ = 14.3 ms^−1^ and (b) *V*_*Initial*_ = 6.3 ms^−1^.

Figure 8 displays several temporal consecutive patterns of saliva droplets’ distribution, which are described by their size for a complete period. A significant portion of space is exposed to pollution during case (I) (*V*_*Initial*_ = 14.3 ms^−1^). This figure also highlights the impact of the environmental parameters, in comparison to the cases in Fig. 4, and for different described phenomena, such as drifting, elongation, disintegration, evaporation, and rotations. By assessing the droplet diameter distribution here, and as discussed in Figs. 5 and 8, it can be concluded that the effect of the gravity and inertia forces on the small saliva droplets (*D*_*droplet*_ ≈ ≤40 *μ*m) as compared to the influence of the indoor airflow is negligible. Medium (50 ≈ ≤*D*_*droplet*_ ≈ ≤150 *μ*m) and large (*D*_*droplet*_ ≈ ≥200 *μ*m) sizes are also more affected by the gravity and inertia forces, respectively.

**FIG. 9.**
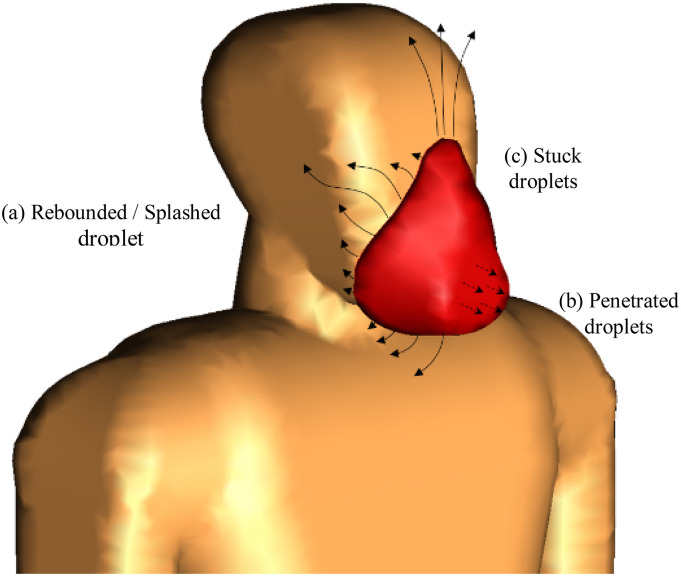
Different modes of interaction between the mask and droplets.

**FIG. 10.**
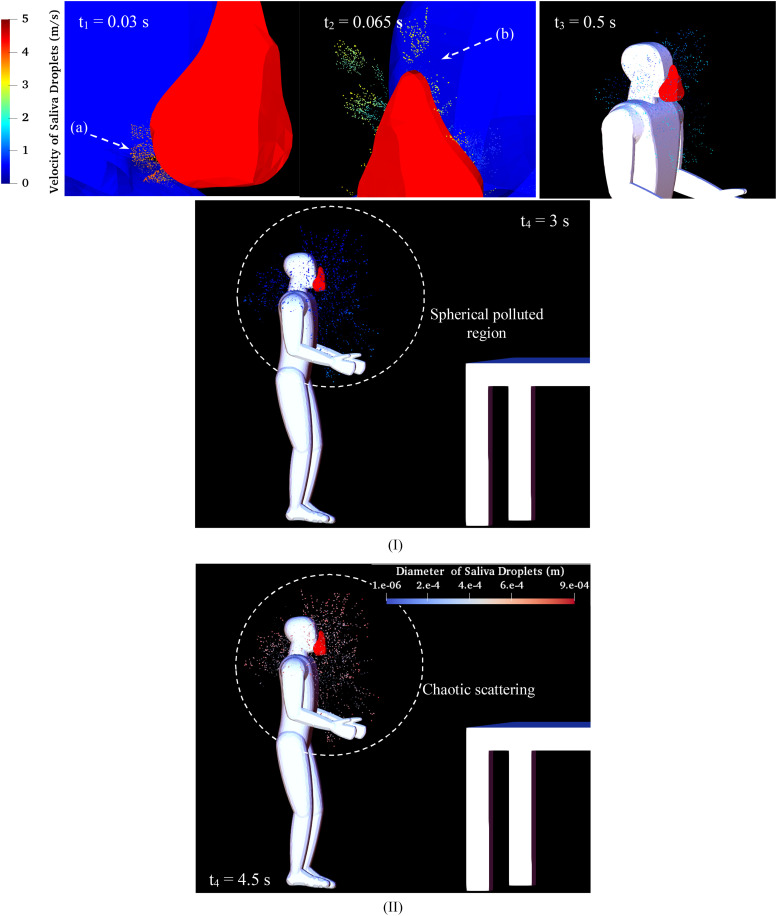
Sneeze over one cycle with the mask: (I) saliva droplet velocity and (II) diameter (*D*_*average*_ = 360 *μ*m, *V*_*Initial*_ = 14.3 ms^−1^).

As mentioned in the literature reviews about micro-droplet dynamics, face mask-wearing is a common effective way for mitigating the respiratory infections. Other methods, such as hand washing, fogging machines, ventilation, and consideration of social distancing, are also beneficial (as mentioned in Figs. 4 and 5). Figure 9 provides a schematic representation of the leakage trajectory of saliva droplets across the face mask. There are three distinct types of local interactions between the saliva droplets and the mask, which can be named rebound/splash, stick, and penetration. A limited number of droplets escape from any opening area due to increasing pressure and velocity originating on the turbulent jet during cough, rebound, or splash. Therefore, face mask tight-fitting is a critical factor that affects mask performance, as indicated in Fig. 9. The bulk of droplets, particularly the larger ones, stick to the fibrous layers of the mask. A few particles with a very small size, lower than the critical droplet diameter, leak from the cover and penetrate. This critical diameter is based on the mask microstructure and varies between 27.19 *µ*m and 146.6 *µ*m, as reported by Leonas *et al.* (2003). Porosity, roughness, and fibrous microstructure can be counted as elements of the mask microstructure (Dbouk and Drikakis, 2020b). In the present simulations, based on the considered initial size distribution of droplets, the number of the penetrated droplets can be ignored, as compared to the total droplet number that is expelled from the mouth. Figure 10 shows that the face mask can prevent airborne pathogens from spreading. “Forward driven droplet” protection by filtering and trapping virus-laden is almost entirely successful, but “outward driven droplet” protection is not so effective, particularly around the edges of the mask and surrounding. The velocity of the large particles that are able to transmit the coronavirus is not significant enough to pass from the mask during sneezing (*V*_*Initial*_ = 14.3 ms^−1^). The saliva micro-droplets rebound and escape from any opening area, especially the pores under the mask (a) and the area above the nose (b) during the hard sneeze [as shown in Fig. 10(I)]. During the high rate of sneezing, the droplets’ scattering region is limited to a sphere with a diameter of around 0.6 m when wearing the face mask instead of traveling the distance of 3.5 m in the naked face case. Figure 10(II) shows the size values for micro-droplets during a sneeze while wearing a face mask. The scattering of the droplets indicates that the droplet size is considerably reduced as compared to the case without using a mask {Fig. 5 [case (III)]}. The Sauter Mean Diameter (SMD) is decreased from *D*_32_ = 205 *μ*m to *D*_32_ = 155 *μ*m for the case with and without using a mask, respectively, in the time *t* = 1 s after sneezing. The sticking of droplets onto the mask surface and their breaking up can be considered as the main reason for this reduction.

**FIG. 11.**
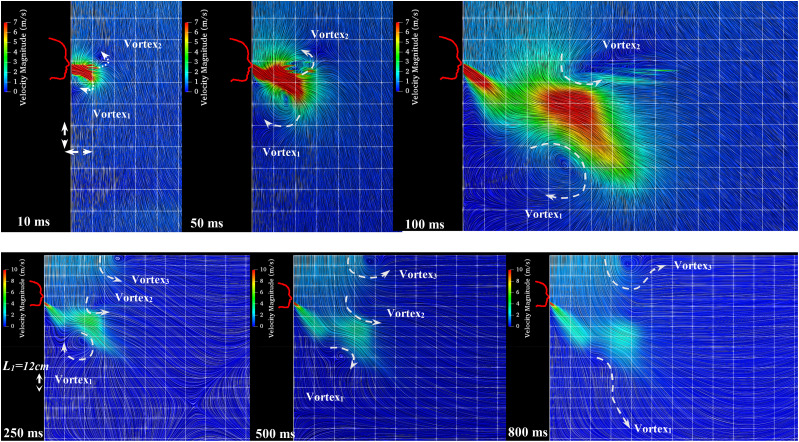
The interaction between the two phases of gas (air) and fluid (saliva). The arrows illustrate the apparent circulation within the cloud.

The dispersion in the sneezing process is affected by the air–mucous, fragmentation of the liquid droplets, and the turbulence of the jet, especially close to the mouth. Figure 11 reveals the airflow with velocity streamlines’ tracing being visualized by the LIC (Line Integral Convolution) model. The vortical structure grows exactly near the mouth, like a source of the infected person, and moves away from the head. The micro-droplet speed exceeds the mean circular air velocity of the vortices, and these vortices affect the patterns of the suspended micro-droplets. Two apparent kinks emitted from the mouth affect the flow streamlines (air outlet combined with droplets), having an important effect on the micro-droplets’ trajectory of the sneeze saliva. A circular changing pattern within the droplet cloud is obvious and has been shown with a pair of arrows during the passing time. Vortex 3, which is caused by the air conditioner, has an effect on the overall flow pattern of sneeze and can control it.

**FIG. 12.**
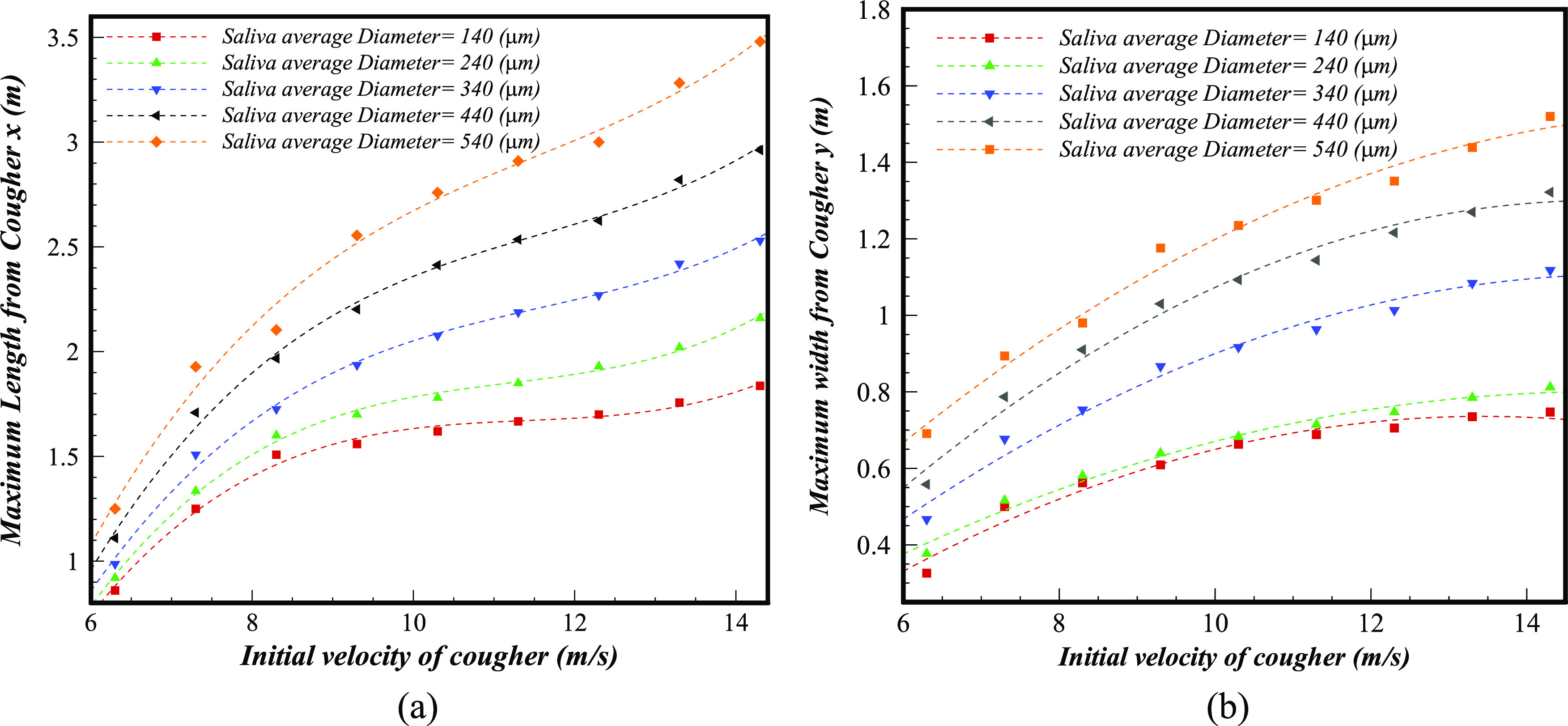
The maximum fall out distance, contamination risk distances, for a broad range of droplet sizes and velocities (*D*_*mean*_ = 90 *μ*m−540 *μ*m, *V*_*Initial*_ = 6.3 ms^−1^–14.3 ms^−1^): maximum (a) length and (b) width (*θ*_*inj*_ = 33°, *Mouth area* = 314 mm^2^).

The penetrated liquid traveling distance is a significant factor that defines the maximum distance reached by saliva droplets. Figure 12 compares the saliva droplets’ maximum fall out length and width, in other words, contamination distances, for a broad range of initial velocities from *V*_*Initial*_ = 6.3 ms^−1^ to 14.3 ms^−1^ and for a wide range of micro-droplet distributions in size (*D*_*mean*_ = 90 *μ*m−540 *μ*m). Figure 12(a) shows that the maximum fall out distance considerably depends on the combination of the average droplet size distribution and the initial velocity of a sneeze. The maximum length and width of the sneeze cloud reach almost 3.5 m and 1.5 m, respectively, at the *V*_*Initial*_ = 14.3 ms^−1^ and *D*_*mean*_ = 540 *μ*m. However, the maximum length and width of the sneeze at the same initial velocity, but at a smaller average size of *D*_*mean*_ = 140 *μ*m, are 1.5 m and 0.73 m, respectively. At the normal sneeze velocities *V*_*Initial*_ = 6.3 ms^−1^, the maximum fall out length and width for the average size of *D*_*mean*_ = 140 *μ*m and *D*_*mean*_ = 540 *μ*m decrease to 1.27 m, 0.71 m and 0.87, 0.36, respectively. It is obvious that the cloud size and its dynamics play an important role in the maximum contamination area of micro-droplets and significantly affects infection risk indoor. This figure reveals that larger droplets carry a larger number of small viruses and pose increased potential risk of airborne propagation diseases, exceeding a safe social distance of 2 m during the roughly mild sneeze.

Based on the numerical simulation of around 45 multiple sneezes with different operational conditions, we deduced polynomial correlations for the maximum length and width of sneezes as follows:

$$L_{\max}=a{V_{Initial}}^{3}-b{V_{Initial}}^{2}+cV_{Initial}-c_{0},$$ (13)

$$W_{\max}=-a{V_{Initial}}^{2}+bV_{Initial}-c_{0}.$$ (14)

Although many parameters are effective in extracting the maximum fall out correlation, herein, we considered these parameters as constant in order to evaluate the effect of the two main parameters: initial velocity and size distribution of droplets. The coefficients of the above polynomial formulation are provided in Table III.

**TABLE III.** Coefficients of the polynomial equation for the deposited saliva droplet area for the maximum length and width.

D_mean_ (*μ*m)	a	b	c	c_0_
(a) Maximum length				
140	0.0056	0.191	2.180	6.69
240	0.0058	0.196	2.239	6.68
340	0.0050	0.172	2.085	6.51
440	0.0056	0.193	2.350	7.39
540	0.0058	0.201	2.476	7.80
(b) Maximum width				
140	0.0073	0.199	0.586	
240	0.0053	0.158	0.384	
340	0.0074	0.227	0.629	
440	0.0095	0.283	0.807	
540	0.0077	0.256	0.595	

**FIG. 13.**
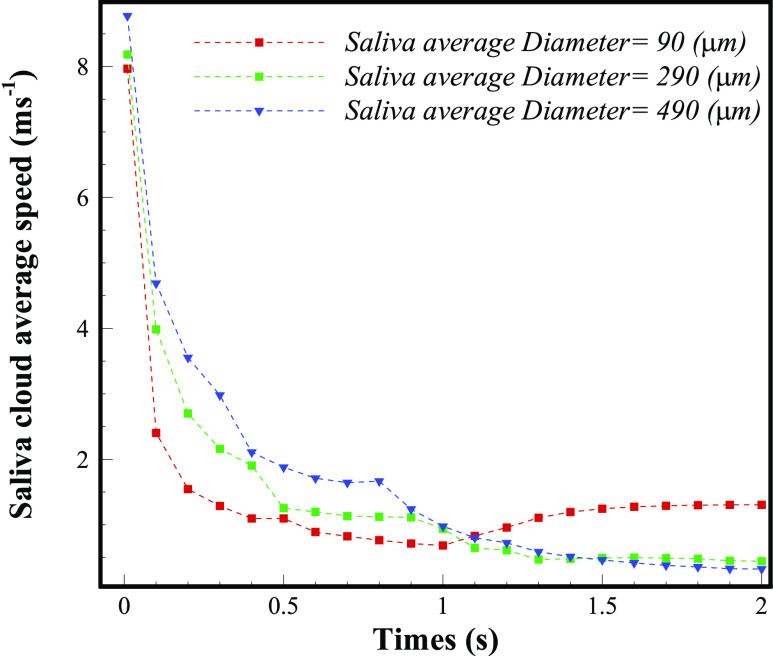
The velocity of the sprayed cloud during a sneeze as compared with various diameters, for a different time, from expelling to fall out (*V*_*Initial*_ = 11.3 ms^−1^).

Figure 13 depicts the decay of the mean velocity of the micro-droplets’ distribution during a sneeze with time from a human mouth. The velocity of micro-droplets, of the varying size range (*D*_*mean*_ = 90 *μ*m−540 *μ*m), is herein measured, and the results are entirely different in terms of fall out time. It can be noted that the droplets of larger size (*D*_*mean*_ = 490 *μ*m) hit the floor in less than 1 s due to higher inertia and gravity and their overall velocity reaches almost zero. However, due to the Brownian movement, drifting, and environmental influence, the smaller size distribution of droplets (*D*_*mean*_ = 90 *μ*m) has not lost all of their velocity, as described before.

**FIG. 14.**
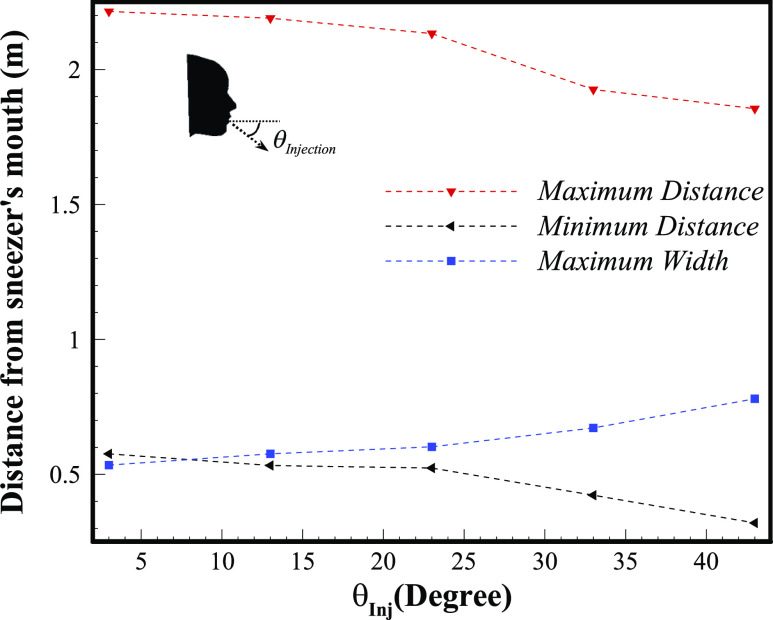
Comparison of various injection angles for a specific sneeze (*V*_*Initial*_ = 14.3 ms^−1^, *D*_*mean*_ = 90 *µ*m).

Figure 14 shows the effect of various horizontal injection angles (${\theta^{○}}_{Injection}=3^{○}-43^{○}$) at a specific sneeze (*V*_*Initial*_ = 14.3 ms^−1^, *D*_*mean*_ = 90 *µ*m) that comes out from a polluting person’s mouth. As a protective action to avoid the spread of respiratory diseases, such as the coronavirus, it is advisable to bend the head during sneezing. In other words, the infected distance declines considerably by increasing the injection angle. The maximum polluted distance drops around 22% from 2.2 m to 1.8 m for sneeze injection angles of 3° and 43°, respectively. The start point of the contaminated area, from the sneezer’s mouth, also decreases from 0.57 m to 0.32 m for the 3° and 43° angles, respectively. However, the maximum width of the contaminated area, as the angle of injection grows, increases slightly (due to higher sneeze power), but it is not as much important as the maximum length in the spreading of the respiratory disease.

**FIG. 15.**
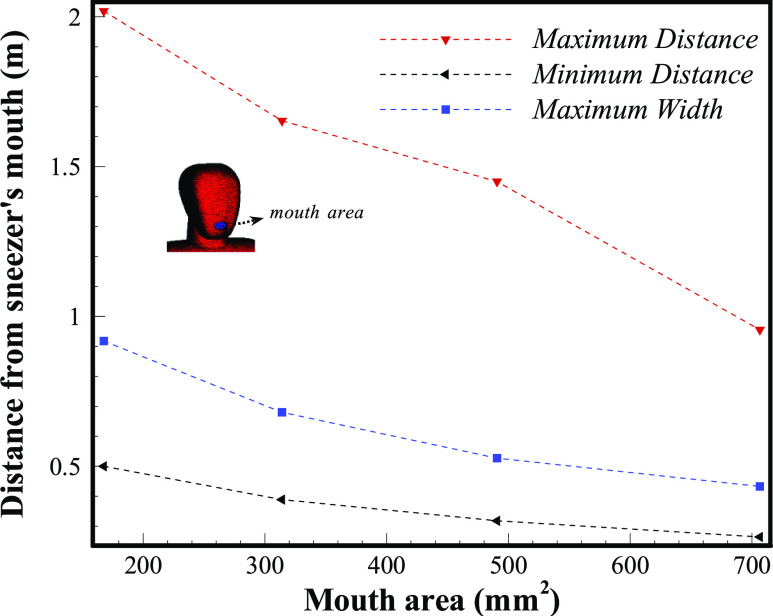
Comparison of various mouth areas for a specific sneeze (*V*_*Initial*_ = 8.3 ms^−1^, *D*_*mean*_ = 290 *µ*m).

Figure 15 demonstrates the impact of different mouth areas during one particular sneeze. For a similar power of sneeze, various people, with different mouth areas, may produce a completely different contaminated area. If one individual continues to open his mouth and the elliptical area of the mouth expands from 170 mm^2^ to 700 mm^2^, the maximum distance that saliva micro-droplets may travel is reduced from 2 m to 0.9 m, respectively.

## V. CONCLUSION

The main goal of the current work is to perform a detailed analysis of the transport characteristics and related fluid dynamics for saliva droplets occurring due to a sneeze in an indoor environment. The following topics were discussed in detail: (a) identifying the micro-droplet transmission mechanisms that are expelled during sneezing within the respiratory tract; (b) characterizing the expelled micro-droplet, including size distribution and velocity, in order to mimic the experimental conditions; (c) comparing the effect of the different size-ranges of micro-droplets and its influence on the transmission routes and deposition pattern; (d) determining a safe settling area based on information from a hundred cases with various initial velocities (*V*_*Initial*_), micro-droplet size distributions (*D*_*p*_), injection angles (*θ*_*Inj*_), and mouth opening areas; (e) further quantifying external factors such as air conditioners and the flow created by a window and a door in the room; and (f) considering various social distancing positions—face-to-face, meeting standing, and near equipment.

The following remarkable findings were identified:

1. We deduced polynomial correlations for the maximum length and width of the contaminated area by considering various sneeze conditions.
2. Sneezing at *V*_*Initial*_ = 22.3 ms^−1^, with an average size of *D*_*mean*_ = 90 *μ*m, caused the saliva droplets to be transported at a distance around 2.3 m, but larger droplets *D*_*mean*_ = 540 *μ*m extended at an even larger length of more than 4 m.
3. Evaluating various horizontal sneezing angles revealed that a full bending of our head, used as a protective action, reduces the droplets traveling distance by more than 22%.
4. The saliva droplet dispersion analysis confirmed that face mask-wearing, due to sneezing, is a very effective protective measure against the spreading of an infectious disease. With this, the maximum transmission area of the droplets is a sphere with a diameter of 0.6 m, and this corresponds to about one-third of the distance a droplet traveled by a naked face.
5. Standing opposite to polluters, face-to face is more susceptible to infection as compared to other positions. The full discharge of a hard sneeze, and its deposit on a surface, takes around 3 s.
6. The effect of gravity and inertia forces on small saliva droplets (*D*_*droplet*_ ≈ ≤40 *μ*m), in comparison to the influence of the indoor airflow, is negligible. Medium (50 ≈ ≤*D*_*droplet*_ ≈ ≤150 *μ*m) and large (*D*_*droplet*_ ≈ ≥200 *μ*m) sized saliva droplets are more affected by the gravity and inertia forces, respectively.
7. Our results indicate that the 2 m social distance may not suffice, since it will depend on the environmental conditions. To improve safety, this distance should be increased to around 4 m.
8. During the sneeze of people with various mouth sizes, the contamination area is different and can experience a 50% increment.
9. The saliva droplet deposition pattern is extremely dependent on the initial size distribution that can be circular, elliptical, and chaotic shape with a corresponding reduction in the size distribution, respectively.
10. Transportation of the saliva droplets is accelerated by the presence of turbulence in the mouth air jet. In addition, appropriate ventilation can effectively control the direction of saliva-disease-carrier and provide a healthy indoor environment.
11. Shorter people are at higher risk of facial contact, since their faces are located on the trajectory of the falling micro-droplets.

## DATA AVAILABILITY

The data that support the findings of this study are available on request from the authors.
